# Sirtuin 1 reduces hyaluronan synthase 2 expression by inhibiting nuclear translocation of NF-κB and expression of the long-noncoding RNA HAS2–AS1

**DOI:** 10.1074/jbc.RA119.011982

**Published:** 2020-01-13

**Authors:** Ilaria Caon, Barbara Bartolini, Paola Moretto, Arianna Parnigoni, Elena Caravà, Daiana L. Vitale, Laura Alaniz, Manuela Viola, Evgenia Karousou, Giancarlo De Luca, Vincent C. Hascall, Alberto Passi, Davide Vigetti

**Affiliations:** ‡ Department of Medicine and Surgery, University of Insubria via J. H. Dunant 5, 21100 Varese, Italy; § Laboratorio de Microambiente Tumoral, Centro de Investigaciones Básicas y Aplicadas (CIBA), Universidad Nacional del Noroeste de la Pcia. de Bs. As., Centro de Investigaciones y Transferencia del Noroeste de la Pcia. de Bs. As. (CIT NOBA UNNOBA-CONICET), B6000, Junín, Argentina; ¶ Lerner Research Institute, ND20, Department of Biomedical Engineering, Cleveland Clinic, Cleveland, Ohio 44195

**Keywords:** hyaluronan, sirtuin 1 (SIRT1), long-noncoding RNA (long ncRNA, lncRNA), inflammation, extracellular matrix, epigenetics, metabolic regulation, glycosaminoglycan, sirtuin, HAS2, HAS2–AS1, SMC

## Abstract

Hyaluronan (HA) is one of the most prevalent glycosaminoglycans of the vascular extracellular matrix (ECM). Abnormal HA accumulation within blood vessel walls is associated with tissue inflammation and is prominent in most vascular pathological conditions such as atherosclerosis and restenosis. Hyaluronan synthase 2 (HAS2) is the main hyaluronan synthase enzyme involved in HA synthesis and uses cytosolic UDP-glucuronic acid and UDP-GlcNAc as substrates. The synthesis of UDP-glucuronic acid can alter the NAD^+^/NADH ratio via the enzyme UDP-glucose dehydrogenase, which oxidizes the alcohol group at C6 to the COO^−^ group. Here, we show that HAS2 expression can be modulated by sirtuin 1 (SIRT1), the master metabolic sensor of the cell, belonging to the class of NAD^+^-dependent deacetylases. Our results revealed the following. 1) Treatments of human aortic smooth muscle cells (AoSMCs) with SIRT1 activators (SRT1720 and resveratrol) inhibit both HAS2 expression and accumulation of pericellular HA coats. 2) Tumor necrosis factor α (TNFα) induced HA-mediated monocyte adhesion and AoSMC migration, whereas SIRT1 activation prevented immune cell recruitment and cell motility by reducing the expression levels of the receptor for HA-mediated motility, RHAMM, and the HA-binding protein TNF-stimulated gene 6 protein (TSG6). 3) SIRT1 activation prevented nuclear translocation of NF-κB (p65), which, in turn, reduced the levels of HAS2–AS1, a long-noncoding RNA that epigenetically controls HAS2 mRNA expression. In conclusion, we demonstrate that both HAS2 expression and HA accumulation by AoSMCs are down-regulated by the metabolic sensor SIRT1.

## Introduction

Hyaluronan (HA)^5^ is a ubiquitous glycosaminoglycan (GAG) that is one of the main components of extracellular matrices (ECMs) (1). It is composed of the repeating disaccharide d-glucuronic acid (GlcNAc), and differently from other GAGs, HA does not possess any chemical modifications (sulfation, acetylation, and epimerization), lacks a protein anchor, and can reach millions of Da of molecular mass (2). In addition to its well-known physical–chemical properties, HA has a critical role in the control of cell behavior, including proliferation, motility, differentiation, and survival (3–9) through the interaction with several receptors, including CD44, RHAMM, and TLR2/4 that are able to trigger specific signaling cascades (10). Moreover, HA can modulate immune systems, and it is generally accepted that low and high molecular mass HA have pro- and anti-inflammatory properties, respectively (11). Furthermore, heavy chains (HC) of inter-α-trypsin inhibitor can be covalently bound to HA, forming HC–HA matrices, through the action of tumor necrosis factor-inducible gene 6 protein (TSG6) (12, 13). Although HC–HA has an essential physiological role during mammalian oocyte maturation (14), in pathological conditions monocytes interact with HC–HA matrices via cell-surface CD44, thereby contributing to immune cells' recruitment to the site of inflammation (15). As HA regulates a variety of cellular phenomena, it is not surprising that it has critical roles in several pathologies, including cancers (16, 17), vascular diseases (18), diabetes (19), and respiratory diseases (20) that are the first causes of death in Western countries.

In tissues, HA has a rapid turnover, and its synthesis is catalyzed by a family of three isoenzymes HAS1, -2, and -3. Conversely, its degradation is due to different hyaluronidases (HYAL1–6) that work at acidic pH, and other related enzymes (*i.e.* TMEM2 and KIAA1199) that have an optimum pH around the physiological value (21, 22). HASes are very unusual proteins as they are transported to the plasma membrane where they are able to form homo- and heterodimers and are activated to extrude the nascent HA chain into the extracellular space (23). In physiological conditions, HAS1 and HAS2 synthesize HA polymers of high molecular mass, whereas HAS3 synthesizes shorter HA polymers (24), and the production of low molecular mass HA can be obtained by the action of degrading enzymes as well as by oxidative stress or UV light (25, 26). Interestingly, the stoichiometry of the cytosolic UDP substrates has a critical role to define HA polymer length, and the C-terminal region of HASes appears to have regulatory functions (27).

At the cellular level, the specific role of each HAS isoenzyme is still unknown, but among the three HASes, HAS2 is considered the most important one. Its genetic deletion leads to early embryonic death due to cardiac defects, whereas the HAS1 and HAS3 knockouts are viable and fertile (28). Interestingly, HAS2 activity is strictly regulated by several mechanisms that can act both at the protein level (as phosphorylation, *O*-linked *N*-acetylglucosamine, and ubiquitination) (29) as well as at the genetic level (30). Another critical point to consider in HAS2 regulation is its intracellular trafficking, as has recently been shown by Melero-Fernandez de Mera *et al.* (31). Several transcription factors are known to modulate HAS2 in response to growth factors, hormones, and cytokines (30). Recently, it has been described that HAS2 antisense 1 (HAS2–AS1), a long-noncoding RNA that belongs to the class of natural antisense transcripts, is able to control HAS2 epigenetically (32) and that HAS2–AS1 is able to alter the chromatin structure around the HAS2 promoter inducing HAS2 transcription in vascular smooth muscle cells (33) and tumor cells (34, 35).

HAS substrates are cytosolic UDP-GlcNAc and UDP-glucuronic acid (UDP-GlcUA), which are synthesized by UDP-glucose (UDP-Glc) dehydrogenase (UGDH). We previously showed that UGDH overexpression and silencing led to an increase and a decrease of the HAS2 transcript, respectively, suggesting a regulatory mechanism involving cytosolic UDP-Glc that is able to coordinate the expression of HAS2 with the presence of its substrate (36). Interestingly, UGDH catalyzes the double oxidation of the C6 of UDP-Glc converting the alcoholic group into a carboxylic group by using two molecules of the cofactor NAD^+^ that are converted to NADH. Therefore, the synthesis of UDP-GlcUA is able to influence the NAD^+^/NADH ratio (32).

NAD^+^ controls the activity of several enzymes, including sirtuins (37). Sirtuin 1 (SIRT1) belongs to the family of NAD^+^-dependent deacetylases, and its protective role in cancers, vascular diseases, and aging is well-known (38–40). It is generally accepted that when nutrients are not limiting, NAD^+^ levels are low, although when there is nutrient shortage or caloric restriction NAD^+^ increases and activates sirtuins (41). SIRT1 is located in both the nucleus, where it can deacetylate histones, and in the cytosol, where it can deacetylate several proteins, including RelA/p65 that inhibits NF-κB activation (42, 43). Interestingly, SIRT1 controls critical aspects of vascular SMC biology and pathology, including differentiation (44) and calcification (45).

As HAS2 is a critical enzyme involved in atherosclerosis with vessel thickening and its transcription is regulated by NF-κB (46), this study investigated whether HAS2 expression could be regulated by SIRT1 in human aortic smooth muscle cells and whether SIRT1 could control pro-atherogenic behavior of AoSMCs after TNFα proinflammatory treatments.

## Results

### HAS2 is the main enzyme involved in HA synthesis in AoSMCs

**Figure 1.**
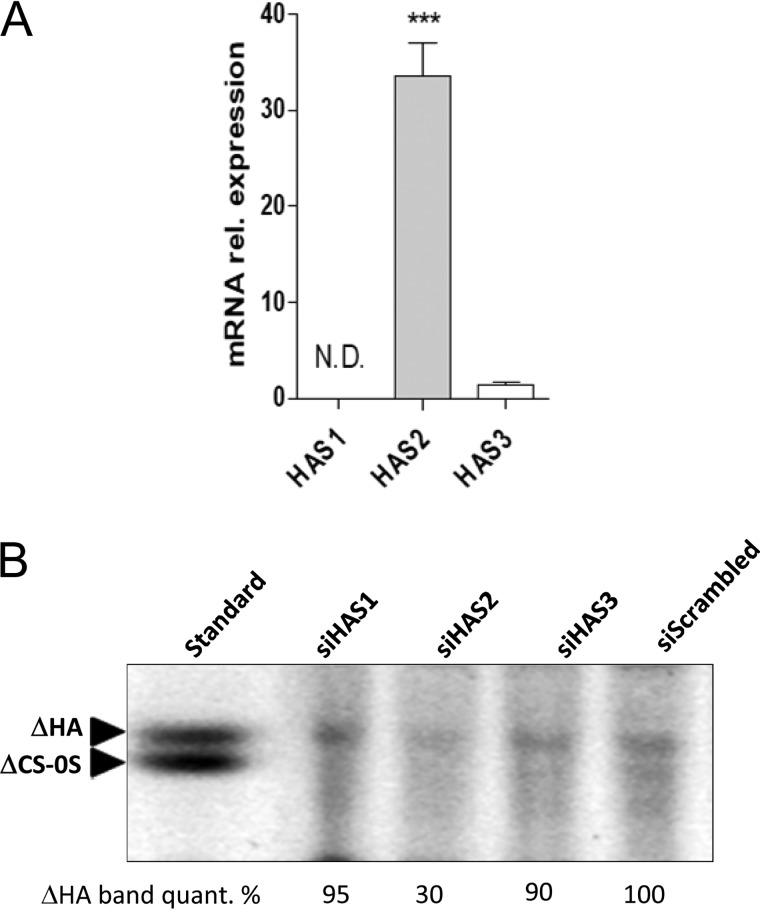
**HASes expression and HA synthesis in AoSMCs.** *A,* quantitative RT-PCR analyses are shown for basal HASes mRNA levels in AoSMCs. Data are expressed as mean ± S.E. of three independent experiments. ***, *p* < 0.001; *N.D.*, not determined. *B,* image is shown of a representative PAGEFS analysis of ΔHA and ΔCS-0S disaccharides in the culture medium of AoSMCs after the silencing of HASes. AoSMCs were transfected with 50 nm of scrambled siRNA (siScrambled) or transfected with 50 nm of each HAS siRNA. After 48 h, cell culture media were collected, and ΔHA and ΔCS-0S were analyzed by PAGEFS. The quantification of the bands was done by measuring their optical density using the ImageJ software.

AoSMCs generally produce high amounts of HA, which is the main component of vascular ECM, along with type I and III fibrillar collagen, elastin, and versican (47). Gene expression analysis of AoSMCs showed that HAS2 is the prevalent HAS isoform with mRNA levels 30 times higher than HAS3, whereas HAS1 expression was not detected (Fig. 1*A*). To determine the contribution of each isoform on HA synthesis, AoSMCs were nucleofected with 50 nm siRNA against the three HAS isoforms, and after 48 h of incubation, the amounts of HA secreted in cell culture media were analyzed by PAGEFS (Fig. 1*B*) (48). The electrophoretic analysis showed that the silencing of HAS2 caused a 70% decrease in the levels of unsaturated HA disaccharides (ΔHA), whereas the knockdown of HAS3 and HAS1 reduced HA disaccharides amount by only 5 and 10%, respectively, providing evidence for a pivotal role of HAS2 in AoSMC HA synthesis. No effects were detected on the band corresponding to the unsaturated chondroitin 0–sulfate disaccharide (ΔCS-0S). Considering the importance of HAS2 with respect to HAS1 and HAS3, our study focused primarily on HAS2 expression.

### SIRT1 activators inhibit HAS2 expression and reduce pericellular HA matrix

**Figure 2.**
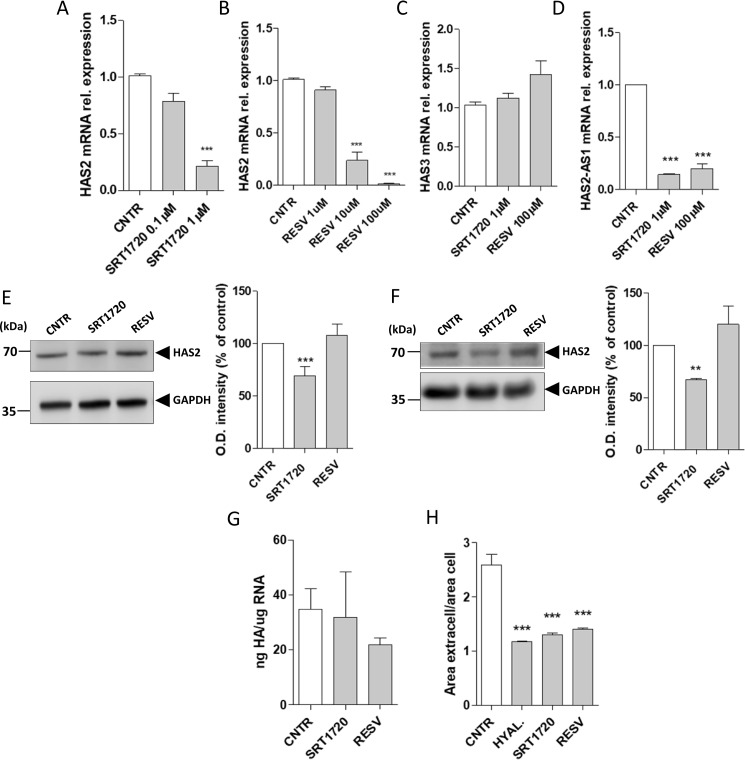
**Effects of SRT1720 and RESV on HAS2 expression and HA production.** Quantitative RT-PCR analyses are shown for HAS2 expression in AoSMCs treated for 24 h with 0.1 or 1 μm SRT1720 (*A*) and 1, 10, or 100 μm RESV (*B*). Data are reported as mean ± S.E. of three independent experiments. ***, *p* < 0.001. Quantitative RT-PCR of HAS3 (*C*) and HAS2–AS1 (*D*) expression after AoSMCs were treated with 1 μm SRT1720 or 100 μm RESV for 24 h. Data are reported as mean ± S.E. of three independent experiments. ***, *p* < 0.001. Western blotting analyses and relative quantification (*bar graph*) are show for a 30-μg protein extract from AoSMCs treated with 1 μm SRT1720 or 100 μm RESV for 24 h (*E*) and for 48 h (*F*). The images report a representative immunoblot for HAS2 and GAPDH upon the treatment with 1 μm SRT1720 or 100 μm RESV. *Numbers* represent the molecular mass of the relative protein expressed in kDa. The analysis was performed measuring the optical density of the bands, and values are expressed as percentage variation of the control ± S.E. of four independent experiments. **, *p* < 0.01, and ***, *p* < 0.001. *G,* quantification of HA amounts determined by ELISA-like assays is shown for AoSMCs cultured in media after a 24-h treatment with 1 μm SRT1720 or 100 μm RESV. Results are represented as nanograms of HA normalized to total micrograms of RNA of three independent experiments. *H,* pericellular coat areas were determined by particle exclusion assay. As a control, AoSMCs were incubated for 1 h with 2 units/ml of HYAL from *S. hyaluroliticus*. Results are expressed as the ratio between the area of the ECM and the area of the cells. Experiments were conducted four times, and HA pericellular coat values are reported as mean ± S.E. ***, *p* < 0.001; *CNTR*, control.

To investigate the effects of SIRT1 on HAS2 expression, we treated AoSMCs for 24 h with resveratrol (RESV), an antioxidant polyphenol nonflavonoid compound that is able to indirectly activate SIRT1 (49), and with SRT1720, a selective synthetic SIRT1 activator (50, 51). The exposure to SRT1720 showed a dose-response effect on HAS2 mRNA expression, with a stronger inhibitory effect at the concentration of 1 μm (Fig. 2*A*). A similar trend was observed with increasing amounts of RESV, where the concentration of 100 μm was the most efficient to decrease HAS2 mRNA levels to near zero (Fig. 2*B*). Considering the effects on HAS2 transcript, all the following experiments used 1 μm SRT1720 and 100 μm RESV. At these concentrations, both the compounds did not show any effects on cells viability (data not shown). The effects of SRT1720 and RESV were specific on HAS2 mRNA as these compounds failed to modulate HAS3 transcript levels (Fig. 2*C*). As HAS2 expression can be regulated by HAS2–AS1, we found that SRT1720 and RESV were also able to inhibit the expression of HAS2–AS1 by 85% (SRT1720) and 80% (RESV) (Fig. 2*D*), confirming the co-regulation of HAS2 and HAS2–AS1 in these cells (33). Western blotting experiments showed that SRT1720 reduced HAS2 protein ∼30% after 24 and 48 h, whereas RESV did not have any effects on HAS2 protein levels at 24 and 48 h of treatment (Fig. 2, *E* and *F*).

As HAS2 is the most important enzyme in AoSMCs for the production of HA, we measured HA levels after the treatment with SRT1720 and RESV and did not find significant changes in the levels of secreted HA in the culture medium (Fig. 2*G*). However, both SRT1720 and RESV caused significant reductions of the pericellular coats of AoSMCs (Fig. 2*H* and in Fig. S1) comparable with the treatment with HYAL, suggesting that the pericellular matrix of AoSMCs is predominantly composed of HA, which can be stimulated by the two SIRT1 activators.

### SIRT1 regulates HAS2 expression in AoSMCs

**Figure 3.**
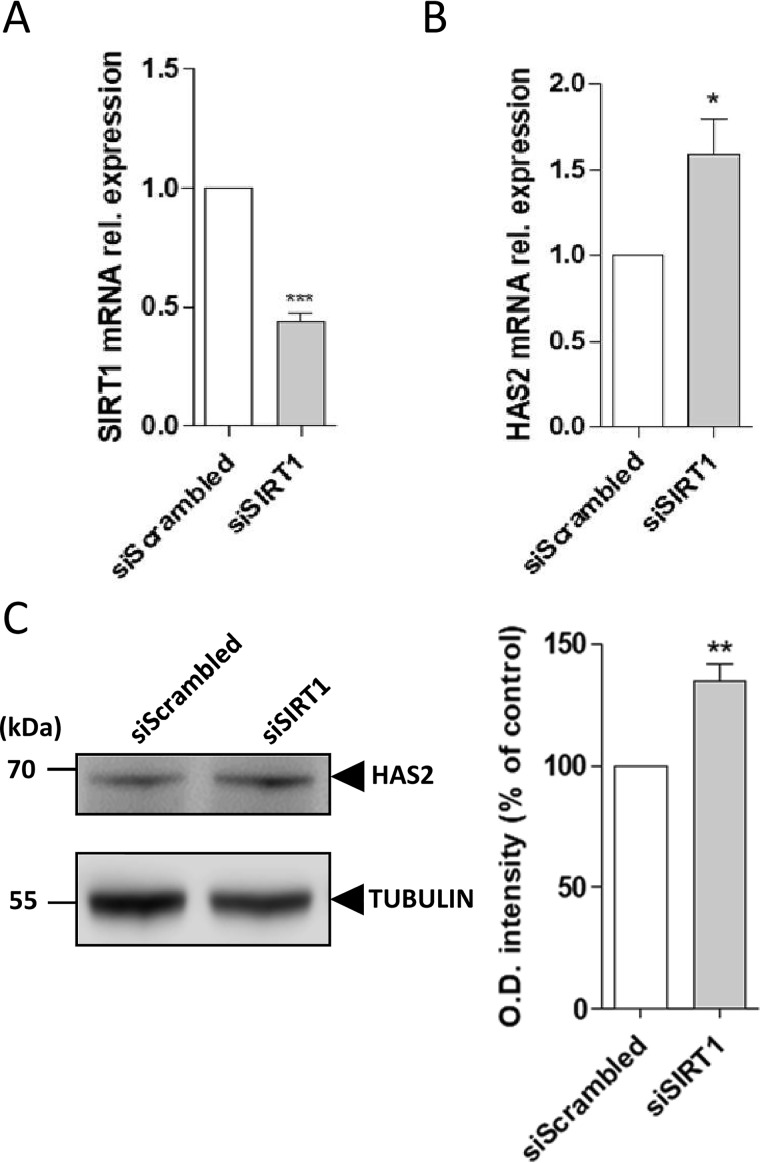
**SIRT1 regulates HAS2 expression in AoSMCs.** Quantitative RT-PCR analyses show SIRT1-silencing efficiency (*A*) and HAS2 mRNA expression after transfection of 100 nm siRNA against SIRT1 in AoSMCs (*B*). Experiments were done three times and are displayed as mean ± S.E. *, *p* < 0.05, and ***, *p* < 0.001. *C,* HAS2 protein levels were analyzed by Western blottings of AoSMC lysates after 100 nm siSIRT1 transfection, and *bars* show relative quantification. Images represent one experimental replicate of the immunoreactive bands for HAS2 and α-tubulin in the different experimental conditions. *Numbers on the margin* of the blots represent the molecular masses expressed in kDa. The analysis was performed measuring the optical density of the bands, and values are expressed as percentage variation of the control of four independent experiments. **, *p* < 0.01.

To confirm whether SIRT1 could regulate HAS2 expression, we transfected AoSMCs with an siRNA against SIRT1, which silenced SIRT1 ∼60% (Fig. 3*A*). The silencing of SIRT1 caused an ∼60% increment of HAS2 mRNA expression with respect to the control (Fig. 3*B*). Moreover, Western blot analysis using a HAS2-specific polyclonal antibody showed the presence of an immunoreactive band at 63 kDa, and the relative densitometric quantification revealed a 30% increase of the HAS2 protein levels (Fig. 3*C*), indicating that SIRT1 was able to regulate both HAS2 mRNA and protein expressions.

### SIRT1 activators protect AoSMCs from inflammation

**Figure 4.**
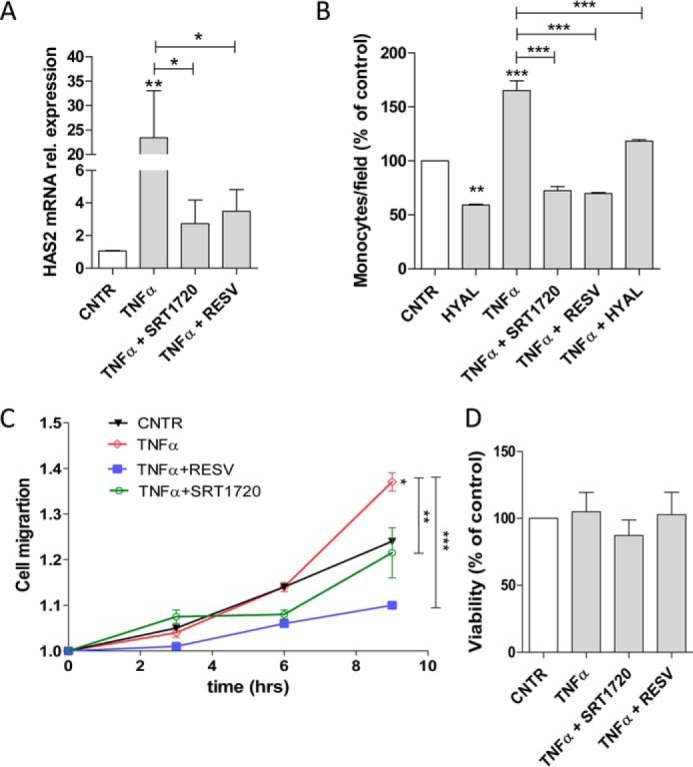
**Effects of SIRT1 activators on TNFα-induced inflammation.** *A,* HAS2 mRNA levels were determined by quantitative RT-PCR in AoSMCs treated for 24 h with 0.1 μg/ml TNFα alone or in combination with 1 μm SRT1720 or 100 μm RESV. Experiments were conducted five times, and data are represented as gene relative expression ± S.E. *, *p* < 0.05; **, *p* < 0.01. *B,* U937 monocyte adhesion assays were performed on AoSMCs treated for 24 h with TNFα alone or in combination with 1 μm SRT1720 or 100 μm RESV. Experiments were conducted three times, and data are expressed as percentage of control ± S.E. **, *p* < 0.01; ***, *p* < 0.001. *C,* AoSMC migration was measured by scratch assay after treatment with 0.1 μg/ml TNFα alone or in combination with 1 μm SRT1720 or 100 μm RESV. Cell migration was calculated by analyzing the scratch area at different time points (0, 3, 6, and 9 h) normalized to the starting scratched area values. **, *p* < 0.01; ***, *p* < 0.001. *D,* AoSMC viability was determined by MTT assay after 24 h of treatment with 0.1 μg/ml TNFα alone or with 1 μm SRT1720 or 100 μm RESV. Values are expressed as mean ± S.E. of three independent experiments performed in quadruplicate. *CNTR*, control.

Recent studies have shown that SRT1720 and RESV have anti-inflammatory effects and exert a protective role in atherosclerosis (52, 53). To mimic the inflammatory status of AoSMCs during atherosclerosis, we treated the cells with TNFα, a pleiotropic cytokine that mediates vascular SMC migration and proliferation (54, 55). Fig. 4*A* shows that stimulation with 0.1 μg/ml TNFα greatly increased the HAS2 mRNA level. Interestingly, the treatment of SRT1720 or RESV together with TNFα restored HAS2 mRNA almost to control levels, indicating the ability of SIRT1 to modulate HAS2 expression and confirming the powerful anti-inflammatory effect of the compounds used. As inflammation can induce monocyte binding to HA (56, 57), we evaluated the ability of U937 monocytes to bind to HA produced by AoSMCs. The exposure to TNFα enhanced the adhesion of U937 monocytes ∼60% to AoSMCs (Fig. 4*B* and Fig. S3), which was reduced to control levels by HYAL. Interestingly, the simultaneous treatment of 0.1 μg/ml TNFα with 1 μm SRT1720 or 100 μm RESV decreased the number of adherent monocytes below control levels (Fig. 4*B* and Fig. S2) similar to the digestion with HYAL alone. This suggests that some monocyte binding in the control and the TNFα plus HYAL cultures may be independent of HA.

A critical event during vessel wall thickening is TNFα-mediated migration of AoSMCs to neointima (58). We therefore performed a scratch assay to evaluate the effects of SRT1720 and RESV on AoSMC motility (Fig. 4*C* and Fig. S3). The exposure of AoSMCs to 0.1 μg/ml TNFα significantly increased cell motility with respect to the control, whereas the simultaneous treatment with TNFα and SRT1720 or RESV reduced the closure of the scratch, suggesting that such compounds can directly influence AoSMC migration counteracting the pro-inflammatory stimulus of TNFα. However, TNFα and the activators of SIRT1 did not alter AoSMC proliferation and viability (Fig. 4*D*).

### SIRT1 activators modulate HA receptor expression and HA-modifying enzymes

**Figure 5.**
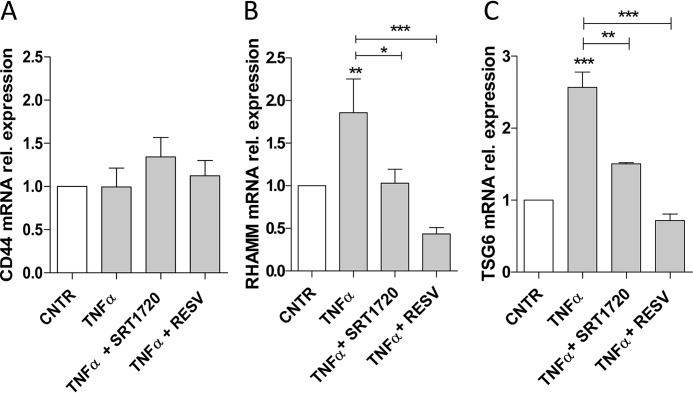
**SIRT1 activators modulate expression of HA receptors and HA-modifying enzymes.** Quantitative RT-PCR experiments show CD44 (*A*), RHAMM (*B*), and TSG6 (*C*) mRNA levels. Gene expressions were analyzed in AoSMCs treated for 24 h with 0.1 μg/ml TNFα alone or with 1 μm SRT1720 or 100 μm RESV. Data are represented as mean ± S.E. of four independent experiments. *, *p* < 0.05; **, *p* < 0.01; *** *p* < 0.001; *CNTR*, control.

HA triggers several cellular responses interacting with different HA receptors as well as with the modifying enzyme TSG6 (15). RHAMM is the main receptor mediating AoSMC motility during vascular injury (59). As shown in Fig. 5*B*, TNFα enhanced the expression of RHAMM, whereas the addition of SRT1720 and RESV significantly decreased RHAMM mRNA levels (Fig. 5*B*). Notably, these treatments did not significantly change CD44 mRNA expression (Fig. 5*A*), the main HA receptor involved in the regulation of vascular SMC proliferation and viability (60).

Furthermore, we studied the expression of TSG6, which has a critical role in immune cells and HA recognition in pericellular HC–HA coats (15). Fig. 5*C* shows that treatment with 0.1 μg/ml TNFα induced a 2.5-fold increase of TSG6 mRNA expression, whereas the simultaneous addition of 1 μm SRT1720 or 100 μm RESV significantly reduced the TSG6 messenger.

These results suggest that SIRT1 activators modulate the expression of the critical proteins involved in HA-mediated motility and inflammation, likely by formation of a monocyte adhesive HC–HA matrix.

### SIRT1 activators reduce natural antisense transcript HAS2–AS1 expression and inhibit NF-κB nuclear translocation

**Figure 6.**
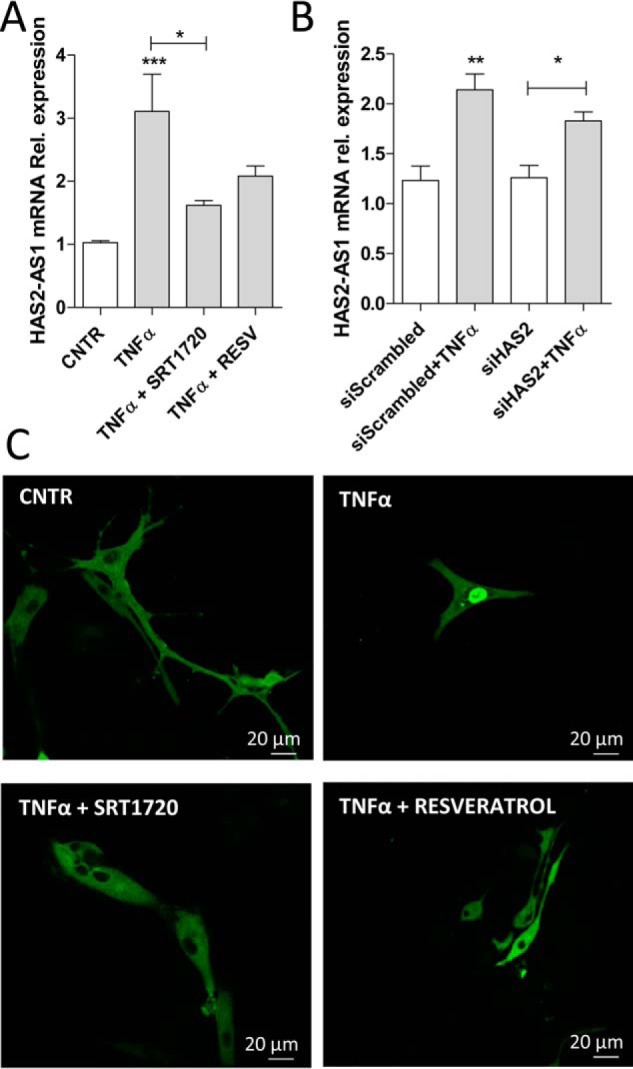
**SIRT1 activators affect HAS2–AS1 expression and NF-κB nuclear translocation.** *A,* quantitative RT-PCR analyses are shown for HAS2–AS1 expression in AoSMCs treated for 24 h with 0.1 μg/ml TNFα alone or with 1 μm SRT1720 or 100 μm RESV. Data are expressed as relative expression of HAS2–AS1 with respect to its control (*CNTR*). Values are reported as mean ± S.E. of three independent experiments performed in duplicates. *, *p* < 0.05; **, *p* < 0.01; ***, *p* < 0.001. *B,* quantitative RT-PCR analyses are shown for AoSMCs nucleofected with 50 nm scrambled siRNA or HAS2 siRNA and left untreated or treated with 0.1 μg/ml TNFα for 24 h. Data are shown as mean ± S.E. of three independent experiments. *, *p* < 0.05; **, *p* < 0.01. *C,* representative images are shown for AoSMCs nucleofected with 4 μg of pcDNA3-GFP-RelA plasmid and grown on coverslips. Twenty four hours after the transfection, cells were treated as indicated for 24 h, washed in PBS, and observed by confocal microscopy (×63 objective). *Bars*, 20 μm.

To investigate the molecular mechanism through which SIRT1 activators inhibit HAS2 expression, we studied HAS2–AS1, one of the main regulators of HAS2 transcription in AoSMCs (33). Quantitative RT-PCR analysis demonstrated that HAS2–AS1 levels increased three times upon the treatment with TNFα (Fig. 6*A*). Interestingly, the simultaneous treatment with TNFα or SRT1720 and RESV significantly reduced HAS2–AS1 expression (Fig. 6*A*).

Control experiments were carried out to exclude a possible involvement of HAS2 in the control of HAS2–AS1 expression. As shown in Fig. 6*B*, silencing HAS2 by siRNA nucleofection did not influence HAS2–AS1 levels. Moreover, HAS2 knockdown did not affect the up-regulation of HAS2–AS1 caused by 0.1 μg/ml TNFα treatment (Fig. 6*B*) thus confirming the critical role of HAS2–AS1 in the regulation of HAS2 expression and not vice versa.

NF-κB is an important mediator of the inflammatory responses triggered by TNFα. The activation of this pathway involves the translocation of the p65 subunit from the cytoplasm to the nucleus and the subsequent transcription of the target genes (61). To visualize the subcellular localization of p65, we nucleofected AoSMCs with a plasmid coding for a GFP–p65 fusion protein. Our results show that the exposure of AoSMCs to 0.1 μg/ml TNFα stimulated the translocation of GFP–p65 from the cytoplasm to the nucleus (Fig. 6*C*), whereas the combined treatment with SRT1720 or RESV retained the subunit in the cytoplasm. These results suggest that the stimulation of SIRT1 can prevent the activation of an NF-κB–signaling pathway.

Control experiments demonstrated that HAS2 silencing did not affect p65 localization (Fig. S5), whereas greatly reduced AoSMC migration after the stimulation with 0.1 μg/ml TNFα confirmed the critical role of HAS2 to sustain cell motility (Fig. S5).

**Figure 7.**
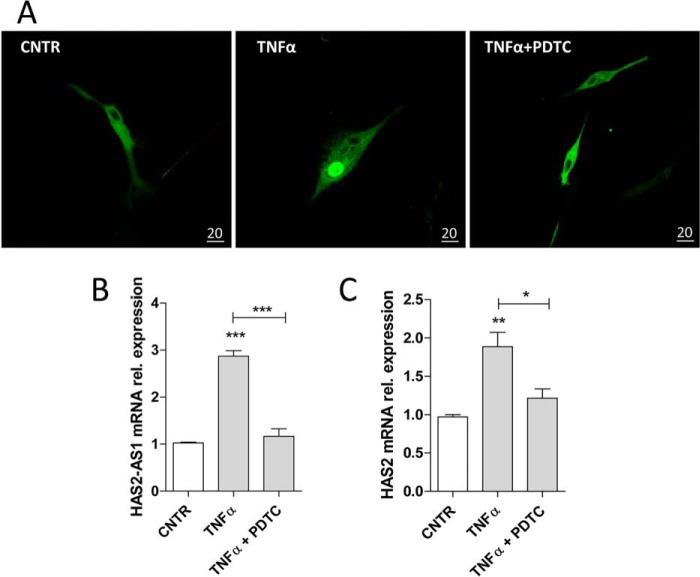
**NF-κB blockade influences HAS2–AS1 expression.** *A*, confocal microscopy images are shown for AoSMCs seeded on coverslips, nucleofected with 4 μg of pcDNA3-GFP-RelA, and treated with 0.1 μg/ml TNFα alone or in combination with 10 μm PDTC (×63 objective). *Bars*, 20 μm. Quantitative RT-PCR analyses are shown for HAS2–AS1 (*B*) and HAS2 (*C*) expression of AoSMCs treated with 0.1 μg/ml TNFα alone or with 10 μm PDTC for 24 h. Data are displayed as mean ± S.E. of three independent experiments. *, *p* < 0.05; **, *p* < 0.01; ***, *p* < 0.001; *CNTR*, control.

To verify the pivotal role of NF-κB in the regulation of HAS2–AS1 in vascular cells, we evaluated p65 localization by treating AoSMCs for 24 h with 10 μm PDTC, a selective inhibitor of IKBα phosphorylation that blocks p65 nuclear translocation (62). Fig. 7*A* shows that AoSMCs exposed simultaneously to PDTC and TNFα retained p65 in the cytoplasm. Moreover, this combined treatment inhibited AoSMC migration (Fig. S6) and significantly decreased the expression of HAS2–AS1 and HAS2 compared with stimulation with TNFα alone (Fig. 7, *C* and *D*). These data confirm the critical role of SIRT1 activators in the regulation of a NF-κB pathway and HAS2–AS1 expression.

## Discussion

In this study, we demonstrate a new regulatory mechanism of HAS2 in vascular SMCs exerted by SIRT1, the yeast sir2 homolog that belongs to the class of NAD^+^-dependent deacetylases. Several studies have shown that the regulation of HA synthesis and the control of HAS2 activity are influenced at multiple levels, including transcriptional, post-translational, and epigenetic regulative mechanisms (23). Interestingly, also the availability of the HA precursor UDP-GluUA is able to influence HA production, and the overexpression of UGDH (the enzyme that converts UDP-Glc into UDP-GlcUA) increased HA levels and HAS2 expression in AoSMCs (36). From a metabolic point of view, this reaction is very important, as it requires two molecules of NAD^+^, suggesting that the synthesis of HA can be a crucial anabolic pathway able to influence the NAD^+^/NADH ratio. Besides synthesis of GAGs, UDP-GlcUA is fundamental for other cellular processes like the reaction of hepatic detoxification (2). Furthermore, the NAD^+^/NADH ratio tightly regulates SIRT1 activity, as NAD^+^ is a critical cofactor for substrate deacetylation. Other metabolic processes able to strongly influence this ratio are the mitochondrial β-oxidation of fatty acid, the conversion of pyruvate into lactate catalyzed by the lactate dehydrogenase, and the NAD salvage pathway (63).

Importantly, the metabolism of HA shares another aspect with SIRT1 activity, the contrasting action involved in cardiovascular diseases. In atherosclerosis, HA exerts a negative role in the onset and the progression of the disease. Under pathological conditions, the ECM of AoSMCs is altered and is mainly composed of HA, which stimulates cell proliferation and migration toward the intimal layer, contributing to vessel thickening (46, 64). Moreover, an overproduction of HA in the genetic background of the apoE-deficient mouse strain promotes atherosclerosis development in the aorta (64). However, the systemic inhibition of HA synthesis by 4-methylumbelliferone showed severe damage in the endothelial cell glycocalyx and promoted the progression of atherosclerosis (65), suggesting that a minimum amount of HA is necessary to maintain the structure of cellular glycocalyces. On the contrary, SIRT1 has been demonstrated to have a protective role in vascular pathologies as it can inhibit neointima formation, atherosclerosis, and vascular SMC hypertrophy (38, 66–68). Thompson *et al.* (69) showed a significant difference in SIRT1 levels from human vascular SMCs isolated from occluded arteries with atherosclerotic lesions compared with nonoccluded sections of the same arteries, confirming that the reduced expression or activity of SIRT1 is a key point in vascular dysfunction.

To investigate the correlation between HAS2, HA production, and SIRT1 activity, we treated AoSMCs with two different SIRT1 activators, SRT1720 and RESV. Although their mechanism of action is still under debate (70), these compounds have been shown to activate SIRT1 in *in vivo* and *in vitro* models (71–73) probably acting like allosteric effectors (74, 75). Indeed, our data show that the exposure of AoSMCs to SRT1720 and RESV did not alter SIRT1 mRNA expression (Fig. S4). This result is in line with the findings of other groups, where the treatment with SRT1720 did not increase SIRT1 expression but caused metabolic effects that required SIRT1 activity (71, 76).

Our results showed that the exposure to SRT1720 decreased HAS2 expression both at the mRNA and protein levels, although the reduction on HAS2 protein was not as efficient as the reduction of HAS2 transcript (probably due to post-translational modifications that alter HAS2 expression and enzymatic activity (77)). However, the selective silencing of SIRT1 increased HAS2 mRNA and protein levels. Interestingly, the treatment with RESV inhibited HAS2 mRNA but not the protein production, suggesting that SIRT1 may have different effects on HAS2 protein. Indeed, SRT1720 and RESV are structurally unrelated, and SRT1720 is hundreds of times more efficient than RESV to activate SIRT1 (78). Moreover, the activation of SIRT1 by SRT1720 and RESV reduced the amount of pericellular HA, suggesting that SIRT1 could be able to regulate HAS2 expression and hence HA production. As the ability of vascular SMCs to migrate and proliferate to the intima is sustained by pro-inflammatory cytokines (79), we treated AoSMCs with TNFα to mimic a pro-inflammatory microenvironment *in vitro*.

Our data demonstrate that treatment with TNFα alone induced AoSMC migration, increased the adhesion of monocytes to the pericellular HA, and stimulated HAS2 production. The changes observed in cell motility are supported by the modulation of RHAMM, the HA receptor that specifically regulates vascular SMC migration (59). The up-regulation of TSG6 upon TNFα exposure could explain the formation of an HC–HA-enriched pericellular coat that is adhesive for monocytes. Interestingly, SRT1720 and RESV counteracted the TNFα effects, reducing RHAMM expression and AoSMC motility, as well as the levels of TSG6 and monocyte adhesion.

The stimulation with TNFα without or with SRT1720 or RESV did not influence AoSMC proliferation and viability. This result is in line with the lack of CD44 modulation, as CD44 is one of the main regulators of vascular SMC proliferation (60, 80).

All these data strongly support the hypothesis that HA is one of the main players to trigger SMC motility and to induce immune cell recruitment. SIRT1 activation, through the inhibition of HA metabolism, can reduce the effects of a proinflammatory microenvironment on vascular cells *in vitro*.

To investigate the mechanism that allows the regulation of HAS2 by SIRT1, we examined the antagonistic cross-talk between SIRT1 and NF-κB. NF-κB is the major transcription factor that mediates TNFα-induced inflammatory responses through the nuclear translocation of its subunit p65. It is known that SIRT1 mediates deacetylation of p65 thereby preventing its translocation into the nucleus, even upon the stimulation with RESV or SRT1720 (81–83). Moreover, the acetylation/deacetylation balance is important to regulate nuclear trafficking. Indeed, some sirtuins (*i.e.* SIRT7 and SIRT2) can control the nuclear export of NF-κB by deacetylating the small GTPase Ran (84, 85). Alternatively, modifications in NF-κB shuttling could be due to an impairment of the nuclear transport proteins, such as importin α5 (86). Although we did not investigate the acetylation status of p65, our results revealed that the treatment with TNFα stimulated p65 nuclear translocation in AoSMCs, whereas the activation of SIRT1 retained p65 in the cytoplasm, confirming the inhibitory effect of SIRT1 on the TNFα-induced inflammatory response.

**Figure 8.**
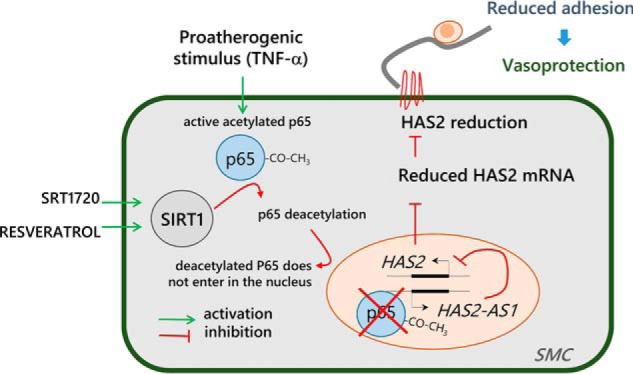
**Working model for protective effects of SIRT1 via HAS2–AS1 and NF-κB.** Stimulation of AoSMCs with TNFα can activate the p65 subunit of NF-κB through acetylation with its consequent translocation into the nucleus and the activation of target genes involved in the inflammatory response. In the nucleus, p65 is able to activate HAS2–AS1 promoter, which induces HAS2 expression and subsequent synthesis of a monocyte adhesive extracellular HA matrix on AoSMCs. The activation of SIRT1 by SRT1720 and RESV initiates the inhibition pathway (*red*) by retaining p65 in the cytoplasm, probably by promoting deacetylation of p65, which prevents p65 nuclear translocation that prevents activation of an HAS2–AS1 promoter. This would inhibit HAS2 mRNA and protein expression, thereby preventing formation of the monocyte adhesive HA matrix, immune cells recruitment, and AoSMC migration, thus protecting AoSMCs from TNFα-induced inflammation.

Previous studies revealed the presence of NF-κB–binding sites on the HAS2–AS1 promoter (33, 87), which could explain the increase of HAS2–AS1 mRNA levels after TNFα stimulation. According to our working model (Fig. 8), the pro-inflammatory stimulus of TNFα causes p65 nuclear translocation and the subsequent activation of the HAS2–AS1 promoter, which in turn stimulates HAS2 expression. In contrast, the activation of SIRT1 by SRT1720 and RESV (although at different extents) inhibited p65 nuclear translocation, HAS2–AS1 promoter activation, and therefore HAS2 expression. As HAS2–AS1 has specific effects on chromatin structure around the HAS2 promoter (32), it is not surprising that all the treatments have strong effects on HAS2 rather than on HAS3 expression. HAS2–AS1 exhibited a coordinated expression with HAS2 as already demonstrated by Michael *et al.* (87). To confirm the proposed pathway, the HAS2 silencing did not influence HAS2–AS1 expression or NF-κB localization, whereas the inhibition of NF-κB nuclear translocation prevented both HAS2–AS1 and HAS2 expression in proinflammatory conditions. Therefore, our data support the hypothesis that SIRT1 regulates HAS2 expression via NF-κB and HAS2–AS1.

The partnership between SIRT1 and AMPK is longstanding. AMPK is the main ATP/AMP sensor in mammalian cells activated during calorie restriction and energy stress status. In the literature there are many papers describing that AMPK can function as a SIRT1 activator and that, mutually, SIRT1 is required for AMPK activation (72, 88). The anabolic pathway related to HA synthesis is a high-energy–demanding process, and it is allowed only when all the cellular energetic requirements are satisfied. Indeed, *in vitro* experiments with AoSMCs demonstrated that HAS2 activity and, subsequently, HA production can be inhibited by AMPK through the phosphorylation of HAS2 Thr-110 (89).

Because SIRT1 can function both in the nucleus and in the cytoplasm, the deacetylase activity of SIRT1 can also be addressed to HAS2 protein itself or to the histones regulating chromatin accessibility to the HAS2 promoter. In conclusion, the effects of SIRT1 on HAS2 regulations are widespread and can be the result of a synergic activity of SIRT1, which could act at the nuclear level controlling p65 deacetylation or chromatin accessibility after histone modifications or at cytoplasmic levels activating AMPK or de-acetylating HAS2 protein.

## Experimental procedures

### Cell cultures and treatments

Primary human AoSMCs were purchased from Lonza, grown for 5–8 passages in SMGM2 culture medium supplemented with 5% FBS, and maintained at 37 °C in the presence of 5% CO_2_. Briefly, 1.2 × 10^5^ cells were plated in a 35-mm dish and incubated for 24 h with DMEM (Euroclone) containing 0.2% FBS to induce starvation. After 24 h, DMEM was replaced with complete medium, and cells were treated for 24 h with 1 μm SRT1720 (Selleck Chemicals), 100 μm RESV (Selleck Chemicals), and/or with 0.1 μg/ml TNFα (Abnova) to induce the inflammatory responses. To inhibit p65 nuclear translocation, cells were treated with 10 μm ammonium pyrrolidinedithiocarbamate (PDTC, Sigma) for 24 h.

### Gene expression determinations by quantitative RT-PCR

Total RNA was isolated from AoSMCs with the PureLink^TM^ RNA mini kit (Invitrogen), retrotranscribed using the high-capacity cDNA synthesis kit (Applied Biosystems), and amplified with an ABI Prism 7000 instrument (Applied Biosystems). The following human TaqMan gene expression assays were used: HAS1 (Hs00155410_m1); HAS2 (Hs00193435_m1); HAS3 (Hs00193436_m1); HAS2–AS1 (Hs03309447_m1); SIRT1 (Hs01009006_m1); CD44 (Hs01075861_m1); RHAMM (Hs00234864_m1); TSG6 (Hs00200178_m1); and β-actin (Hs99999903_m1). The relative gene expressions were determined by 2^−ΔΔ*Ct*^ method (90).

### Cell transfection

To knock down SIRT1 expression, 8 × 10^5^ AoSMCs were transiently nucleofected using a Nucleofector Apparatus (Amaxa) and the human AoSMC nucleofection kit (Lonza) with 100 nm SIRT1 siRNA (Silencer Select SIRT1, Ambion) or a scrambled siRNA (silencer negative control 1, Ambion).

The same nucleofection kit was used to silence HASes expressions using 50 nm siRNAs against HAS1 (Silencer® Select HAS1, Ambion), HAS2 (Silencer® Select Pre-Designed HAS2, Ambion), and HAS3 (Silencer® Select HAS3, Ambion). To study GFP–p65 subcellular localization 4 μg of pcDNA3-GFP-RELA plasmid (23255, Addgene) was nuclefected in AoSMCs.

### HA determination

To quantify the amount of HA produced by AoSMCs, cell culture media were collected 24 h after the treatments and diluted 1:100. The quantification of HA was done with the hyaluronan quantikine ELISA kit (R&D Systems) according to the manufacturer's instructions.

The analysis of ΔHA and ΔCS-0S was done by PAGEFS, as described previously (48). Briefly, AoSMC culture medium was digested with 10 milliunits/ml proteinase K (Fynnzymes), and the glycosaminoglycans were purified by ethanol precipitation. The specific unsaturated disaccharides of HA (ΔHA) and ΔCS were obtained by specific glycosidase digestions, derivatized with 2-aminoacridone, and separated by gel electrophoresis.

To evaluate the pericellular coat of HA, a particle exclusion assay was used (91, 92). Briefly, 1.5 × 10^3^ cells were seeded in a 12-well plate and treated with 1 μm SRT1720 and 100 μm RESV. After 24 h, 1.5 × 10^7^ fixed human red blood cells were added to each well. After an incubation time of 30 min at 37 °C, cells were examined by contrast microscopy, and 10 pictures per well were taken. As a control, cells were treated with 2 units/ml *Streptomyces hyalurolyticus* HYAL (Sigma). The analysis of the images and the relative quantification were done using the image analysis software ImageJ.

### Western blotting

Proteins were collected from treated or untreated AoSMCs by using RIPA buffer supplemented with 10% of protease inhibitors (Sigma) and separated on precast 4–12% gradient acrylamide gels (Genscript) at 120 V in 1× Mops Buffer (Genscript). Protein samples were transferred to nitrocellulose membrane at 250 mA for 1 h at 4 °C, blocked in 5% BSA, 0.1% TBS-Tween 20 for 1 h, and incubated with primary antibody overnight at 4 °C. After extensive washing, the membranes were incubated with primary antibody and with horseradish peroxidase–conjugated secondary antibody. Chemiluminescence was detected using the LiteAblot® turbo chemiluminescent substrate (Euroclone), and bands were revealed using the LI-COR Odyssey® IR imaging system (LI-COR Biosciences). Western blotting experiments were done using antibodies against HAS2 (polyclonal Y14, sc34068, Santa Cruz Biotechnology), GAPDH (polyclonal V18, sc20357, Santa Cruz Biotechnology), and α-tubulin (monoclonal 11H10, Cell Signaling).

### Monocyte adhesion assay

Adhesion of U937 monocytes to AoSMC cultures was done as described previously (89, 93). Briefly, 1 × 10^6^ U937 monocytes were resuspended in complete medium, plated on untreated or treated AoSMCs, and incubated for 1 h at room temperature. Subsequently, cells were washed three times with their appropriate medium, and the evaluation of adherent U937 cells was detected by microscopy, counting the number of adherent monocytes in 10 independent fields. To verify that U937 binding was specific to HA, AoSMCs were treated with 2 units/ml of *S. hyalurolyticus* HYAL (Sigma) for 1 h at 37 °C.

### Migration assay

AoSMC migration was determined by a scratch assay (94). Briefly, 1.8 × 10^5^ cells were plated in a 35-mm dish and serum-deprived (0.2% FBS) for 24 h. Three scratches per well were done with a 20-μl pipette tip, and new complete medium was added with 0.1 μg/ml TNFα alone or in combination with 1 μm SRT1720 or 100 μm RESV. Pictures were taken through light microscopy at different time points (0, 3, 6, and 9 h) and analyzed using ImageJ software.

### Cell proliferation assay (MTT)

To study cell proliferation, 5 × 10^3^ AoSMCs were seeded in a 96-well plate and incubated for 24 h with DMEM 0.2% FBS. After 24 h, AoSMCs were treated with 0.1 μg/ml TNFα alone or with 1 μm SRT1720 or 100 μm. At the end of the treatment (24 h), the medium was replaced with 200 μl of fresh SMGM2 culture medium supplemented with 50 μl of 5 mg/ml MTT and incubated at 37 °C for 5 h. The reaction was stopped adding 200 μl of DMSO and 25 μl of Sorensen glycine buffer per well. The plate was read at 570 nm.

### Evaluation of NF-κB nuclear translocation

AoSMCs were nucleofected with the plasmid pcDNA3-GFP-RELA (Addgene) coding for the GFP–p65 protein fusion, plated on poly-l-lysine (100 ng/liter) pre-coated glass coverslips (35 mm in diameter), and treated with 0.1 μm TNFα alone or in combination with 1 μm SRT1720, 100 μm RESV, or 10 μm PDTC (Sigma). After 48 h, cell culture medium was removed, and AoSMCs were washed three times with PBS and fixed with a 4% paraformaldehyde/PBS solution for 10 min at room temperature. Coverslips were mounted on glass slides, and p65 subcellular localization was analyzed by confocal microscopy using a Leica TCS SP5 instrument.

### Statistical analysis

All data are expressed as mean ± S.E. Statistical significance was calculated with Student's *t* test for unpaired data or one-way ANOVA followed by Bonferroni post hoc test. For the migration assays, two-way ANOVA followed by Bonferroni post hoc test analysis were performed. All statistical tests were carried out using GraphPad Prism (GraphPad software, version 5.3, San Diego, CA). All experiments were repeated at least three times (figure legends report the exact number of replicates of each experiments).

## Author contributions

I. C., G. D. L., A. Passi, and D. V. conceptualization; I. C., P. M., D. L. V., M. V., and D. V. formal analysis; I. C., B. B., A. Parnigoni, M. V., and D. V. investigation; I. C., P. M., A. Parnigoni, E. C., and D. L. V. methodology; I. C., B. B., L. A., A. Passi, and D. V. writing-original draft; I. C., V. C. H., A. Passi, and D. V. writing-review and editing; E. K. and A. Passi funding acquisition; A. Passi and D. V. supervision.

## Supplementary Material

Supporting Information

## Footnotes

5 The abbreviations used are:

HA: hyaluronan
AoSMC: primary human aortic smooth muscle cell
ECM: extracellular matrix
GAG: glycosaminoglycan
HAS2: hyaluronan synthase 2
HAS2–AS1: hyaluronan synthase 2 natural antisense 1
HYAL: hyaluronidase
NF-κB: nuclear factor κ–light-chain enhancer of activated B cell
PAGEFS: PAGE of fluorophore-labeled saccharide
RESV: resveratrol
TNFα: tumor necrosis factor α
UDP-GlcUA: UDP-glucuronic acid
MTT: 3-(4,5-dimethylthiazol-2-yl)-2,5-diphenyltetrazolium bromide
PDTC: ammonium pyrrolidinedithiocarbamate
ANOVA: analysis of variance
DMEM: Dulbecco's modified Eagle's medium
FBS: fetal bovine serum
UGDH: UDP-Glc dehydrogenase
HC: heavy chain
SMC: smooth muscle cell
AMPK: AMP-activated protein kinase
CS: chondroitin sulfate
ΔCS-0S: unsaturated chondroitin 0–sulfate disaccharide.
