# Supplementary material for: Sirtuin 1 reduces hyaluronan synthase 2 expression by inhibiting nuclear translocation of NF-κB and expression of the long-noncoding RNA HAS2–AS1
Source: J Biol Chem. 2020 Jan 13;295(11):3485–96. doi: 10.1074/jbc.RA119.011982 (PMC7076221; doi:10.1074/jbc.RA119.011982)
Supplement: Supporting Information [file supp_295_11_3485__index.html]

Sirtuin 1 reduces hyaluronan synthase 2 expression by inhibiting nuclear translocation of NF-kB and expression of the long-non coding RNA HAS2-AS1 — SIRT1 reduces HAS2 expression via NF-kB and HAS2-AS1 — Sirtuin 1 reduces hyaluronan synthase 2 expression by inhibiting nuclear translocation of NF-κB and expression of the long-noncoding RNA HAS2–AS1 — SIRT1 reduces HAS2 expression via NF-κB and HAS2–AS1 — Supporting Information 

# Sirtuin 1 reduces hyaluronan synthase 2 expression by inhibiting nuclear translocation of NF-κB and expression of the long-noncoding RNA HAS2–AS1

## Supporting Information

- Supporting Information (to be published online) - Supporting information - Caon et al.
