# Supplementary material for: Sirtuin 1 reduces hyaluronan synthase 2 expression by inhibiting nuclear translocation of NF-κB and expression of the long-noncoding RNA HAS2–AS1
Source: J Biol Chem. 2020 Jan 13;295(11):3485–96. doi: 10.1074/jbc.RA119.011982 (PMC7076221; doi:10.1074/jbc.RA119.011982)
Supplement: Supporting Information [file supp_RA119.011982_157239_1_supp_457187_q41y7n.pdf]

Supporting Information

Supporting Figure S1

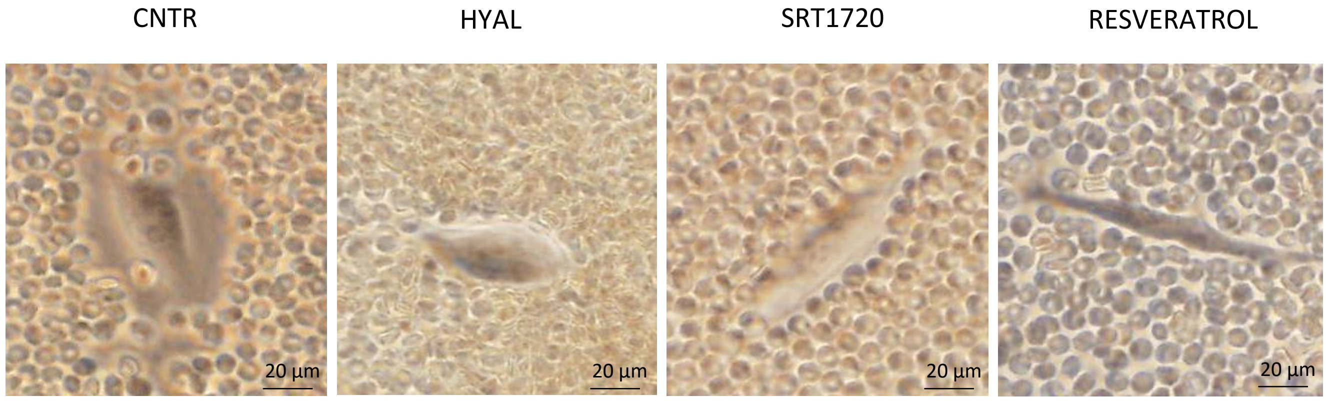

SRT1720 and resveratrol reduce AoSMCs pericellular coats.
Representative images of the particle exclusion assay conducted on AoSMCs treated with 2U/ml hyaluronidase from *S. hyalurolíticus* (HYAL), 1 µM SRT1720 or 100 µM resveratrol.

Supporting Figure S2

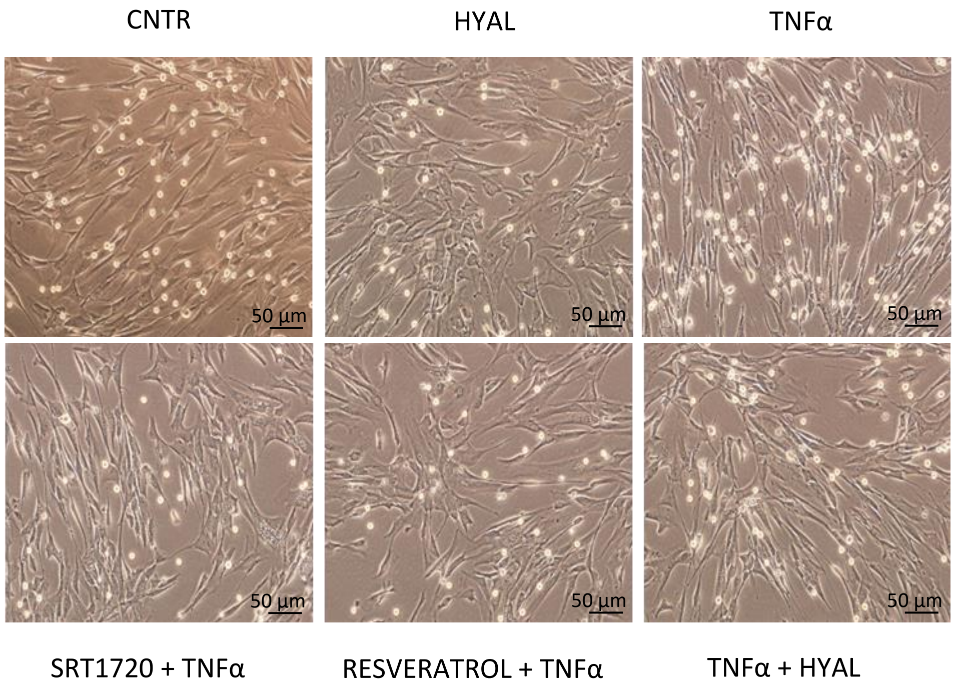

SRT1720 and resveratrol reduce monocyte adhesion after TNFα-induced inflammation.
AoSMCs were treated with 0.1 µg/ml TNFα alone or in combination with 1 µM SRT1720 or 100 µM resveratrol. After 24 hours U937 monocytes were added on AoSMCs and incubated for 30 minutes at room temperature. As a control AoSMCs were treated with 2U/ml hyaluronidase from *S. hyalurolíticus* (HYAL) alone or with 0.1 µg/ml TNFα.

Supporting Figure S3

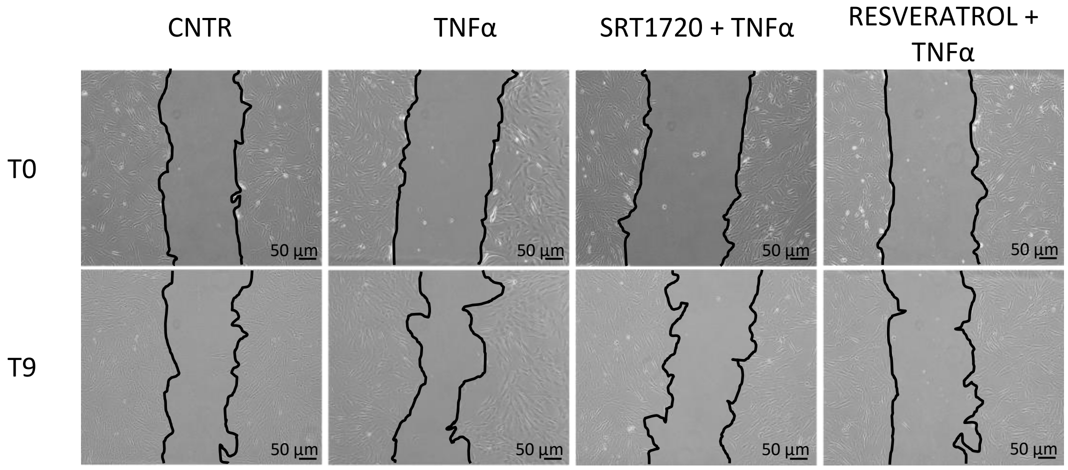

SRT1720 and resveratrol decrease AoSMC migration after TNFα-induced inflammation.
Representative panels from AoSMCs scratch assays after the treatment with 0.1 µg/ml TNFα alone or in combination with 1 µM SRT1720 or 100 µM resveratrol. Pictures display the area of the scratch at 0 and 9 hours after the treatments.

Supporting Figure S4

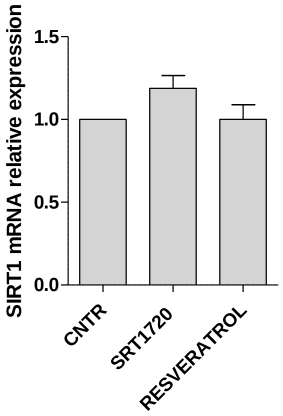

SRT1720 and resveratrol did not alter SIRT1 mRNA levels.
SIRT1 mRNA levels determined by quantitative RT-PCR in AoSMCs treated for 24 hours with 1 µM SRT1720 or 100 µM resveratrol. Data are expressed as mean ± SEM of four independent experiments.

Supporting Figure S5

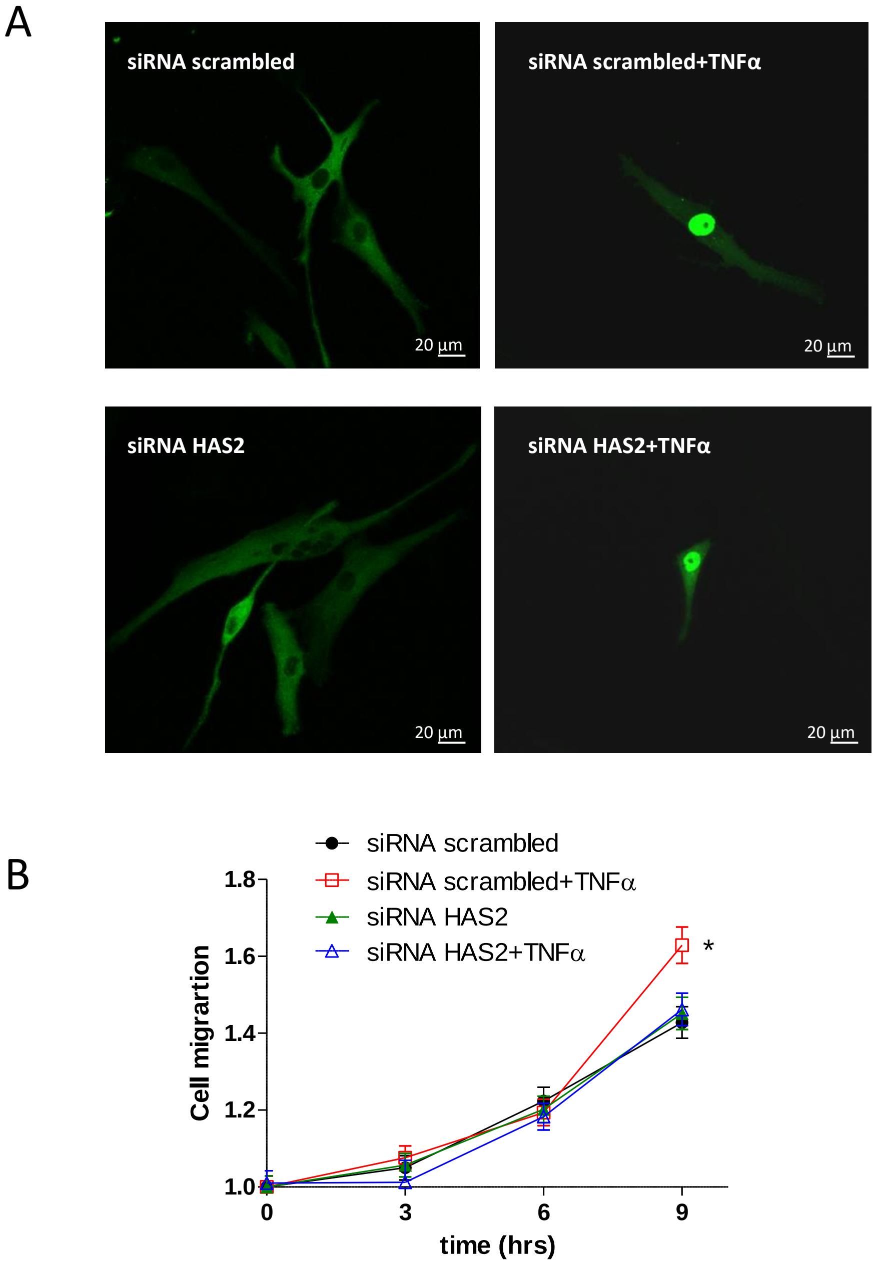

HAS2 silencing does not alter NF-kB localization and AoSMCs migration.

A) Representative images of AoSMCs grown on coverslips and co-transfected by nucleofection with 3 µg of pcDNA3-GFP-RelA and 50 nM siRNA against HAS2 or a scrambled sequence. Twenty-four hours after the transfection, cells were treated with 0.1 µg/ml TNFα for 24 hours and observed by confocal microscopy (x63 objective). Bars 20 µm. B) AoSMCs migration performed by scratch assay after the nucleofection of 50 nM siRNA against HAS2 or a scrambled sequence. One day after the transfection cells were treated with 0.1 µg/ml TNFα for 24 hours. Cell migration was calculated by analyzing the scratch area at different time points (0, 3, 6 and 9 hours) and normalized on the starting scratched area values, * $p<0.05$.

Supporting Figure S6

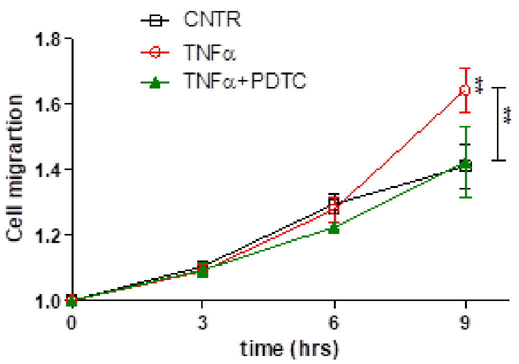

NF-kB blockade inhibits AoSMCs migration.

AoSMCs migration was evaluated by scratch assay. Cells were scratched, washed in PBS and treated with 0.1 µg/ml TNFα alone or with 10 µM PDTC. Cell migration was analyzed comparing the scratch area at different time points (0, 3, 6 and 9 hours) normalized to the starting scratched area values. Data are reported as mean ± SEM of three independent experiments ** $p < 0.01$.
